# Cooperative nucleic acid binding by Poly ADP-ribose polymerase 1

**DOI:** 10.1038/s41598-024-58076-w

**Published:** 2024-03-29

**Authors:** Manana Melikishvili, Michael G. Fried, Yvonne N. Fondufe-Mittendorf

**Affiliations:** 1 Department of Epigenetics, Van Andel Institute, Grand Rapids, MI 49503 USA; 2 Center for Structural Biology, Department of Molecular and Cellular Biochemistry, University of Kentucky, Lexington, KY 40536 USA

**Keywords:** Biochemistry, Molecular biology

## Abstract

Poly (ADP)-ribose polymerase 1 (PARP1) is an abundant nuclear protein well-known for its role in DNA repair yet also participates in DNA replication, transcription, and co-transcriptional splicing, where DNA is undamaged. Thus, binding to undamaged regions in DNA and RNA is likely a part of PARP1’s normal repertoire. Here we describe analyses of PARP1 binding to two short single-stranded DNAs, a single-stranded RNA, and a double stranded DNA. The investigations involved comparing the wild-type (WT) full-length enzyme with mutants lacking the catalytic domain (∆CAT) or zinc fingers 1 and 2 (∆Zn1∆Zn2). All three protein types exhibited monomeric characteristics in solution and formed saturated 2:1 complexes with single-stranded T_20_ and U_20_ oligonucleotides. These complexes formed without accumulation of 1:1 intermediates, a pattern suggestive of positive binding cooperativity. The retention of binding activities by ∆CAT and ∆Zn1∆Zn2 enzymes suggests that neither the catalytic domain nor zinc fingers 1 and 2 are indispensable for cooperative binding. In contrast, when a double stranded 19mer DNA was tested, WT PARP1 formed a 4:1 complex while the ∆Zn1Zn2 mutant binding saturated at 1:1 stoichiometry. These deviations from the 2:1 pattern observed with T_20_ and U_20_ oligonucleotides show that PARP’s binding mechanism can be influenced by the secondary structure of the nucleic acid. Our studies show that PARP1:nucleic acid interactions are strongly dependent on the nucleic acid type and properties, perhaps reflecting PARP1’s ability to respond differently to different nucleic acid ligands in cells. These findings lay a platform for understanding how the functionally versatile PARP1 recognizes diverse oligonucleotides within the realms of chromatin and RNA biology.

## Introduction

Poly (ADP) ribose polymerases (PARPs) are a diverse family of enzymes, with about 18 different protein members in humans. These enzymes are also known as ADP-ribosyl transferases (ARTs) for their ability to transfer ADP-ribose groups to protein substrates or to protein-ADP-ribose adducts (a process called PARylation). PARP1 is the most studied member of this family. PARylation of PARP1 contributes to its role in DNA repair, including repair of single-strand breaks (SSBs) and double-strand breaks (DSBs)^1^, in the stabilization of DNA replication forks^2^ and in the modification of chromatin structure^3^.

**Figure 1:**
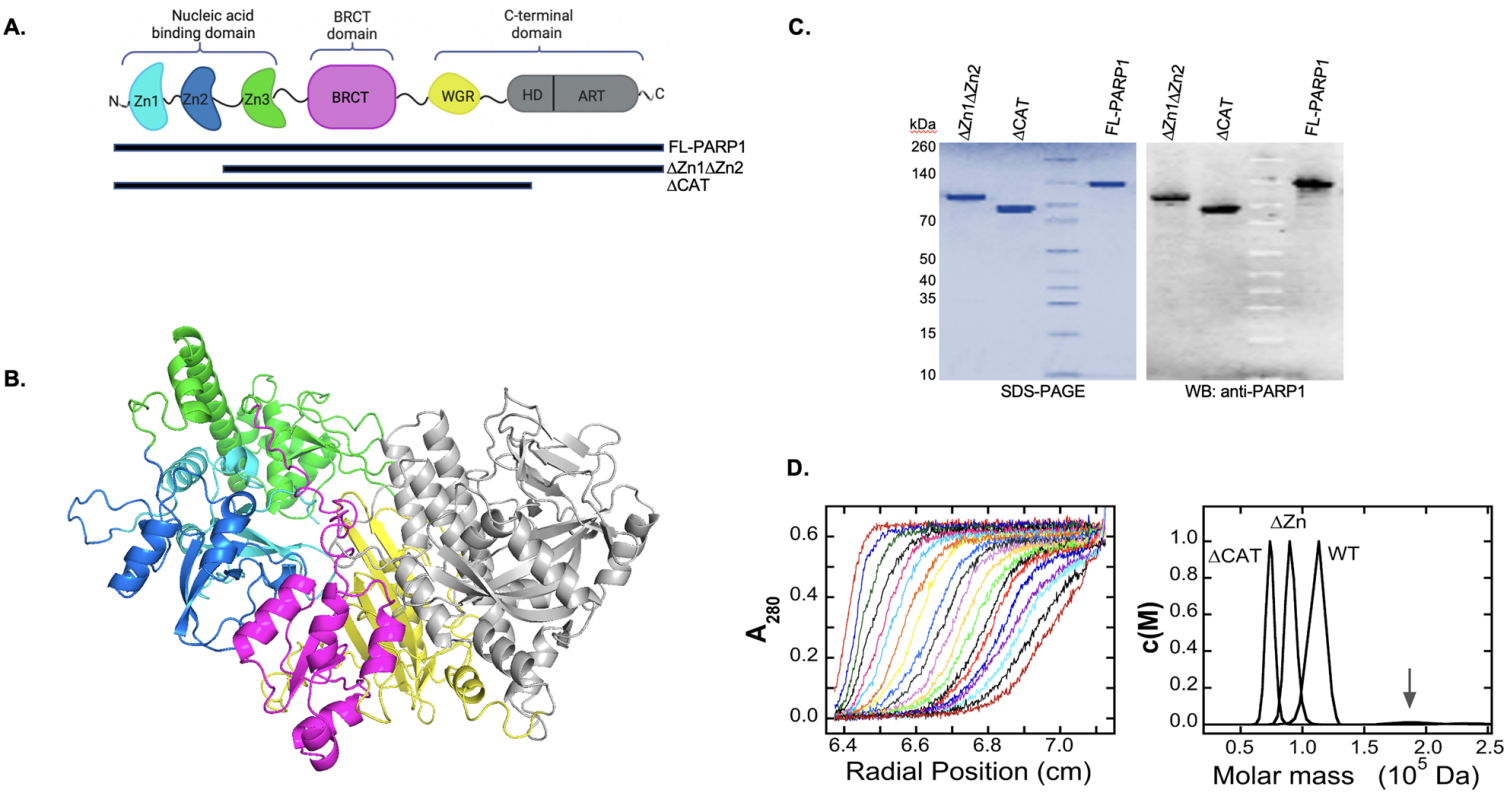
Characterization of PARP1 constructs: A. Top—schematic diagram showing the order and approximate location of the named structural domains. Bottom: schematic showing the different PARP1 constructs used in study. B. Alpha-fold prediction of PARP1 folded structure based on known structures of PARP1 domains bound to DNA^93,94^. C. Gels showing both Coomassie staining and Western blot analysis of the purified PARP1 proteins used in study (complete gel images are found in Supplemental Figure S2). D. Sedimentation velocity analysis of PARP1 proteins. Left: Time evolution of sedimentation for full-length PARP1 protein (4.6 µM) in buffer consisting of 10 mM Tris–HCl, pH 8.0, 100 mM NaCl, 0.1 mM EDTA, 0.1 mM TCEP. Samples were run at 35,000 rpm and 4 °C; absorbance data were acquired at 280 nm. Starting with the left-most curve, each succeeding scan represents a time increment of 5 min. Right: c(M) distributions calculated from numerical fits to the Lamm equation using the program SEDFIT^48–50^. Samples contained full-length PARP1 (4.6 µM; labeled WT), ∆Zn1∆Zn2 mutant PARP1 (6.1 µM; labeled ∆Zn), and ∆CAT PARP1 (7.1 µM, labeled ∆CAT) in the buffer and run conditions described above. Central values of these peaks are consistent with molecular weights predicted for monomers (see Table 2). The arrow indicates the presence of small concentrations of larger species.

PARP1 as a multidomain protein (Fig. 1A and B), contains three main functional domains. The N-terminal part of the protein sequence folds to form three zinc fingers (Zn1, Zn2, Zn3), of which Zn1 and Zn2 are known DNA binding domains and Zn3 is hypothesized to bind RNA^4,5^. The central part of the protein encodes a BRCT(BRCA1 C-terminal)-fold‐containing automodification domain which may also bind DNA^6,7^ while the C-terminal sequences encode domains involved in protein interaction (WGR) and catalysis of ADP-ribose polymerization (CAT;^6,8^). Recently the WGR domain was also implicated in DNA binding^9^. Although Zn1, Zn2, Zn3 and WGR domains have been shown to collaborate in recognizing and binding to DNA strand breaks^10^, how these domains interact with a range of different DNA structures is still subject to debate. For instance, a mutant PARP1 protein containing only Zn1Zn2 domains, bound DNA as a dimer^11,12^ while other protein forms have been reported to bind DNA as a monomer^10,13–15^. Other studies showed that Zn3 homodimerization is not required for DNA-dependent activation of PARP1^11,14,16^. A more recent study using single particle electron microscopy of human PARP1 provided structural evidence of the dimeric structure of PARP1^17^. It is therefore possible that depending on the types of PARP1 structures or substrates PARP1 binds to, it could act as a monomer or a dimer.

The recruitment of PARP1 to DNA damage sites^4,18^ stimulates its catalytic activity, resulting in self-PARylation and PARylation of histones and non-histone proteins^19–21^. A consequence of PARP1 binding and activation is the relaxation of the chromatin structure to allow access by DNA-repair proteins. In transcription, PARP1 competes for binding with the repressive Histone H1, to stimulate gene expression^3,22^. Thus, in both DNA repair and transcription, PARP1 provokes a PAR-mediated recruitment and regulation of chromatin remodeling factors^23–25^, and/or PARylation of histones^24–27^, leading to an open chromatin structure, with subsequent gene expression.

In addition to binding chromatin and DNA, PARP1 also interacts with RNA during co-transcriptional splicing^5,28–31^ and while these studies show that PARP1 can bind a wide range of nucleic acid structures, important features of its binding mechanisms remain to be discovered. To date, the quaternary state of un-PARylated PARP1 is poorly defined, even though this is likely to be the starting state for PARP1 in its regulatory and catalytic interactions^12,17,32^. This is due in some part to the use of truncation mutants or chimeric proteins in studies that could answer this question. The removal of intrinsic domains or the addition of extrinsic ones can have significant effects on quaternary interactions (c.f.,^33,34^). A second question is one of binding mechanism(s). Several studies, including ours (below), give evidence that PARP1 can form multi-protein complexes with nucleic acids^11,12^, while others show single protein binding to isolated sites^6,13–16,32^. Again, in some cases this contrast might be attributed to use of protein truncation mutants that affect binding interactions, or to nucleic acid templates that limit interaction stoichiometry. Finally, while PARP1 has been shown to bind damaged DNA as a monomer^13,14,35,36^, whether it binds undamaged DNA independently as protein monomers or cooperatively has not received enough attention. Our aim in this report is to address these gaps in our knowledge. We have found that under native-like solution conditions, full-length WT-FL-PARP1 and its ∆CAT and ∆Zn1∆Zn2 mutants, sediment as monomers. We show that all three forms are active in binding single stranded and duplex DNAs as well as a single stranded RNA. We determine binding stoichiometries for complexes and find that all proteins tested can form multi-protein complexes and that binding is positively cooperative. In summary, there is a diversity of binding densities and binding site sizes. A full understanding of this diversity will be needed if we are to understand how PARP1 distributes between binding substrates in vivo.

## Materials and methods

### Materials

Reagents. [ $\gamma$^32^P]-ATP was from PerkinElmer; T4 polynucleotide kinase was purchased from New England Biolabs. Electrophoresis grade polyacrylamide was from VWR International. All other chemicals were reagent-grade or better.

### Purification of PARP1 and its derivatives

His-tagged PARP1 expression vectors were a kind gift from the Pascal laboratory (University of Montreal) and purified as previously described (Supplemental Figure S1) and^37^. Briefly, the sequences corresponding to wild-type full-length (WT-FL-PARP1 hence called WT-PARP1, aa 1–1014), ∆CAT-PARP1 (aa 1–662), and ∆Zn1∆Zn2-PARP1 (aa 216–1014) were cloned into the pET28 expression vector. Proteins were expressed in One Shot BL21 (DE3) pLysS competent E. coli cells in the absence of benzamidine and purified by chromatographic fractionations in the following order: (1) Ni–NTA agarose (Qiagen); (2) HiTrap Heparin HP (GE Healthcare), and (3) a gel filtration with Superdex S200 (GE Healthcare). Fractions were monitored by SDS-PAGE and western blot using PARP1 N-terminal and C-terminal antibodies (Active Motif, Carlsbad, CA, USA). Pooled purified fractions (Fig. 1C and Supplemental Figure S2) were concentrated using an Amicon spin concentrator (10,000 Da cut-off, Millipore). The program SEDNTERP (http://www.jphilo.mailway.com/download. htm#SEDNTERP) was used to estimate protein extinction coefficients. This returned values of e_280_ = 1.19 × 10^5^ M^−1^ cm^−1^ for WT-PARP1, e_280_ = 8.43 × 10^4^ M^−1^ cm^−1^ for the ∆CAT protein, and e_280_ = 8.82 × 10^4^ M^−1^ cm^−1^ for the ΔZn1ΔZn2 protein. Protein concentrations were determined by BCA Assay (Thermo Scientific) or by A_280_ using the molar extinction coefficients given above. PARP1 proteins prepared in this way have significant secondary structure as detected by circular dichroism (Supplemental Figure S3) and^5^. Results shown below indicate that these proteins have extended structures expected for native, multi-domain proteins, and that they have specific and distinctive DNA-binding activities.

### Nucleic acids

The sequences of nucleic acids used in this study are given in Table 1. DNAs and RNAs were purchased from Integrated DNA Technologies Company (IDT). Oligonucleotides for use without ^32^P labels were purified by extensive dialysis at 4 °C against 10 mM Tris, 0.1 mM EDTA, pH 8.0. For assays using isotope detection, single-stranded nucleic acids were labeled at 5’ termini with ^32^P as described by Maxam and Gilbert^38^. Labeled oligonucleotides were purified by gel electrophoresis under denaturing conditions (25% polyacrylamide gel, 8 M Urea) and recovered by the crush–soak method^38^, concentrated by extraction with anhydrous n-Butanol and dialyzed at 4 °C against buffer containing 10 mM Tris, 0.1 mM EDTA, pH 8.0. Duplex DNAs were obtained by mixing the top strand (as shown in Table 1) with a 1.05-fold molar excess of unlabeled complement. Nucleic acid concentrations were measured by spectrophotometry at 260 nm, using extinction coefficients provided by the manufacturers. Complementary ssDNAs were heated, cooled to make dsDNAs and visualized on native PAGE (Supplemental Figure S4).

**Table 1:** Sequences of nucleic acids used in this study.

Name	Sequence	Molecular weight (Da)
ds19-mer DNA	5’ – CGT ACG CGG GTT TAA ACG A – 3’	11,617
	3’ – GCA TGC GCC CAA ATT TGC T – 5’	
ss19-mer DNA	5’ – CGT ACG CGG GTT TAA ACG A – 3’	5853
Cy3-T_20_ DNA	5’-Cy3– TTT TTT TTT TTT TTT TTT TT – 3’	6,529
U_20_ RNA	5’ – UUU UUU UUU UUU UUU UUU UU – 3’	6,282

### Sedimentation velocity analyses

Proteins were dialyzed against 10 mM Tris–HCl, pH 8.0, 150 mM NaCl, 0.1 mM EDTA, 0.1 mM TCEP at 4 °C, and the concentration was adjusted to a range of 0.2—1.0 mg/ml. Sedimentation velocity measurements were taken at 4 °C using an AN-60 Ti rotor in a Beckman XL-A analytical ultracentrifuge. Sedimentation coefficient distributions (c(s)), molecular weight distributions (c(M)), and translational friction coefficients (f) were obtained by direct boundary modeling using numerical solutions of the Lamm equation^39^, implemented in the program SEDFIT^40^, obtained from http://www.analytical-ultracentrifugation.com/default.htm. Buffer density and viscosity, and protein partial specific volumes were calculated using SEDNTERP^41^. SEDNTERP was also used to calculate the axial ratios of ellipsoids of revolution from measured translational friction coefficients. SEDNTERP was obtained from http://www.rasmb.bbri.org/.

### Electrophoretic mobility shift assays

Binding reactions were carried out at 20 ± 1 °C in 25 mM Tris (pH 8.0), 150 mM NaCl, 50 mM arginine, 1 mM EDTA, 0.1 mM TCEP, and 1 mg/mL bovine serum albumin. Mixtures were equilibrated at 20 ± 1 °C for 30 min before electrophoresis. Duplicate samples incubated for longer periods gave identical results, indicating that equilibrium had been attained (result not shown). Electrophoresis was carried out in 8% polyacrylamide gels (75:1 acrylamide:bis-acrylamide), containing 90 mM Tris–borate, 2 mM EDTA buffer, pH 8.3^42^. Following electrophoresis, autoradiographic images were captured on storage phosphor screens (GE Healthcare) detected with a Typhoon FLA 9500. Band-quantitation was performed using Image-Quant TL software (GE Healthcare), as described by the manufacturer.

### Quantitative binding analysis

Association constants and cooperativity parameters were evaluated by direct titration of DNA with protein, with binding detected by EMSA. The total concentration of protein binding sites on DNA was always much less than that of the protein allowing the approximation [P]_total_ = [P]_free_ to be used. For the concerted binding of n protein molecules (P) to a single DNA (nP + D $\rightleftarrows$ P_n_D), the apparent association constant is K’ = [P_n_D]/[P]^n^[D]. Here, K’ is the formation constant for the cooperative complex, containing contributions from both protein-DNA and protein–protein interactions. Separating variables and taking natural logarithms gives the following linear relationship.

1 $$\ln \frac{{[P_{n} D]}}{[D]} = n\ln [P] + \ln K^{\prime}.$$

Initial values of [P] were calculated for each titration step using the conservation relation [P] = [P]_tot_ – n[P_n_D], in which [P]_tot_ is the total protein concentration and n is an initial estimate of the stoichiometry. An updated estimate of n was then obtained from the linear dependence of ln[P_n_D]/[D] on ln[P]. The new value of n was fed back into the conservation relation and the cycle iterated until values of n and K’ ceased to change. Ultimately, the slope of the graph yields a value of the stoichiometry n, while at the mid-point of the titration (where ln [P_n_D]/[D] = 0), ln K’ = -n ln [P], allowing evaluation of the formation constant, K’. Values of K’ are difficult to compare when complexes differ in stoichiometry. However, assumption of equipartition of binding free energies allows evaluation of monomer-equivalent association constants, K_mono_ = (K’)^1/n^, which are easier to compare. This approach has been described previously^43,44^.

Cooperativity parameters were evaluated using the McGhee-von Hippel isotherm^45^ as modified by Record et al.^46^ to account for finite lattice size (Eq. 2).

2 $$\frac{\nu }{[P]} = K(1 - s\nu )\left( {\frac{(2\omega - 1)(1 - s\nu ) + \nu - R}{{2(\omega - 1)(1 - s\nu )}}} \right)^{s\, - \,1} \, \left( {\frac{1 - (s + 1)\nu + R}{{2(1 - s\nu )}}} \right)^{2} \left( {\frac{N - s + 1}{N}} \right); where R = \left( {(1 - (s + 1)\nu )^{2} + 4\omega \nu (1 - s\nu )} \right)^{1/2}$$

Here ν is the binding density (protein molecules/nucleotide), calculated from stoichiometry values obtained with Eq. (1). The equilibrium association constant for binding a single site is given by K, the cooperativity parameter by ω, the length of the DNA in base pairs is N, and s is the occluded site size (the size of the site, in base pairs, that one protein molecule occupies to the exclusion of others).

## Results

### PARP1-protein and its derivatives sediment as monomers

Previously we showed that purified full-length PARP1 (WT-PARP1) and the deletion proteins (∆Zn1∆Zn2 and ∆CAT) (Fig. 1A and 1C) have closely similar CD spectra, consistent with the notion that these deletions do not cause large-scale loss of secondary structure^5^. However, these analyses did not reveal the oligomerization states of the proteins, a characterization that is essential for analysis of DNA binding. Sedimentation velocity analyses were performed to fill this gap in our knowledge. Shown in Fig. 1D is the time-evolution of the sedimenting boundary formed by the WT enzyme centrifuged at 4 °C and 35,000 rpm. Also shown are c(M) distributions obtained by sedimentation analysis for all three proteins. All preparations contained single dominant species with molecular weights (MW) in the range 70,000 ≤ MW ≤ 130,000, with no detectible low molecular weight material, and only traces of larger species (indicated by the arrow). The central values and 95% confidence limits of these MW distributions are shown in Table 2, together with monomer molecular weights calculated from amino acid compositions. A comparison of measured and calculated MW values shows that all enzymes sediment as monomers under these solution conditions.

**Table 2:** Comparison of experimental molecular weights of PARP1-proteins with values calculated from amino acid compositions.

Protein	Measured molecular weight^a^	Molecular weight from amino acid composition	f/f_0_	Axial ratio for prolate ellipsoid^b^
WT-PARP1	109,310 ± 5530	113,907	3.01 ± 0.10	9.92 ± 0.91
∆Zn1∆Zn2-PARP1	90,510 ± 4,610	89,805	2.64 ± 0.09	9.62 ± 0.92
∆CAT- PARP1	73,910 ± 3,590	74,522	2.31 ± 0.09	10.25 ± 0.98

^a^Measured by sedimentation velocity analysis. Error ranges represent 95% confidence limits. ^b^Calculated using SEDNTERP^41^.

Sedimentation velocity analysis also returns values of the translational frictional coefficient ratio f/f_0_, where f is the experimentally observed frictional coefficient and f_0_ is that of a sphere of equivalent volume, given by

3 $${f}_{0}=6\pi \eta {\left(\frac{3M\overline{v}}{4\pi {N}_{A}}\right)}^\frac{1}{3}$$

where $\eta$ is the solution viscosity, M the molecular weight, $\overline{v}$ the partial specific volume, and N_A_ the Avogadro’s number. For our proteins, values of f/f_0_ range from 2.31 to 3.01, indicating significant deviation from spherical symmetry (Table 2). Modeled as prolate ellipsoids of revolution, with typical values of protein hydration (0.3 g/g,^47^), these proteins are predicted to have axial ratios in the range 9.6–10.2. Such axial ratios are consistent with elongated structures in which compact domains are flexibly connected by short linkers^10,14^, unlike the AlphaFold prediction shown in Fig. 1B, which likely reflects the compact, DNA-bound PARP1 conformation. Similar values of f/f_0_ and corresponding axial ratios support the notion that the deletions that produce the ∆CAT and ∆Zn1∆Zn2 mutants do not cause large-scale changes in organization or folded state of the resultant proteins.

### Rapidly equilibrating protein complexes are formed with a short, double-stranded DNA

**Figure 2:**
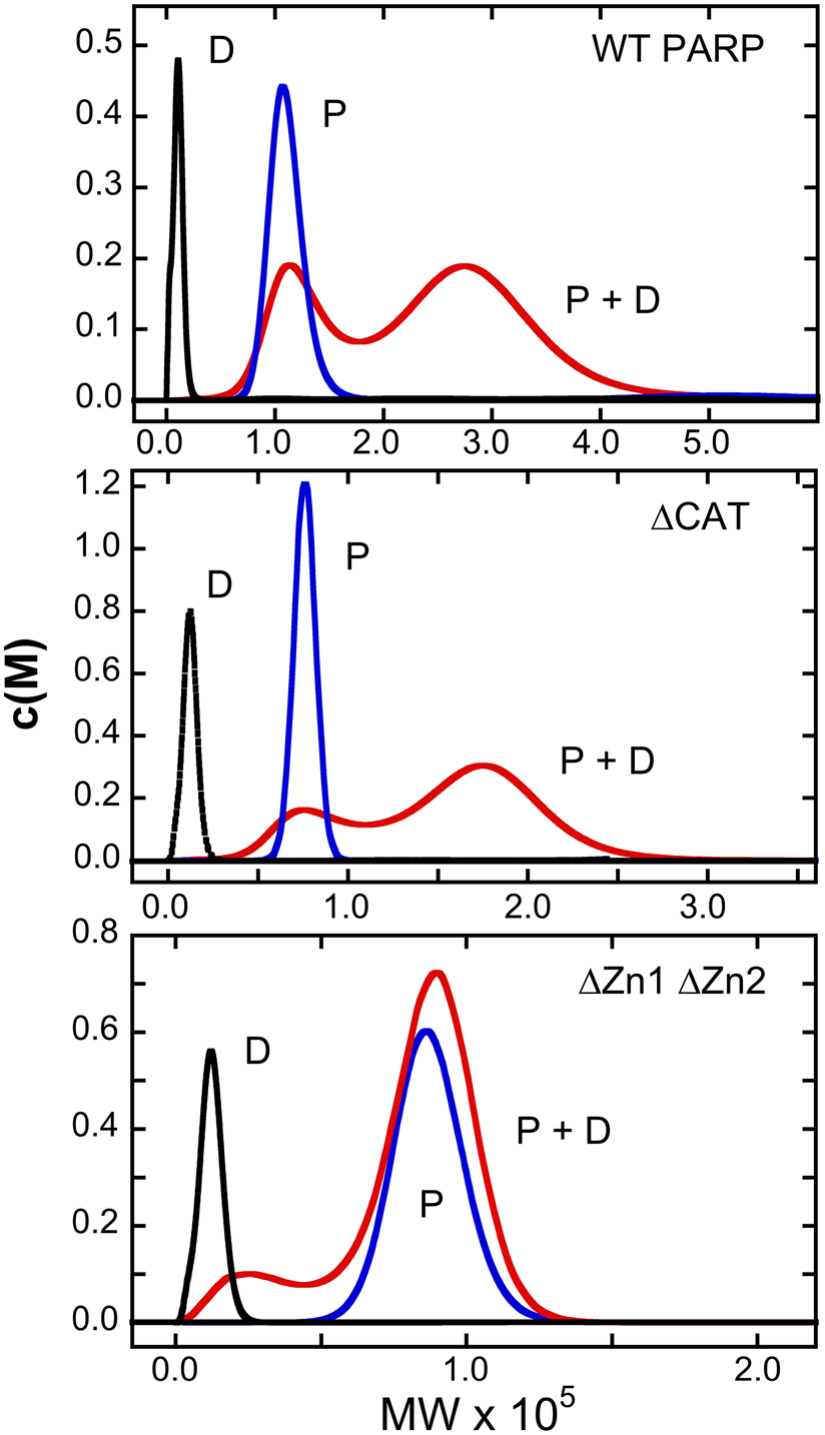
Sedimentation velocity analyses of mixtures containing duplex DNA and PARP1. Samples contained the duplex 19-mer DNA shown in Table 1 or the indicated protein, or a mixture of the two in buffer containing 10 mM Tris–HCl, pH 8.0, 100 mM NaCl, 0.1 mM EDTA, 0.1 mM TCEP. Samples for analysis of WT-PARP1 binding contained 1.6 µM DNA and/or 3.6 µM protein. Samples for analysis of ∆CAT PARP1 binding contained 2.1 µM DNA and/or 9.4 µM protein. Samples for analysis of ∆Zn1∆Zn2 PARP1 binding contained 1.8 µM DNA and/or 7.2 µM protein. All samples were centrifuged at 25,000 rpm and 4 °C; absorbance data were acquired at 260 nm. C(M) distributions^40^ are shown for DNA alone (D), or protein alone (P) or a mixture (P + D).

Addition of a double-stranded 19-mer DNA (sequence shown in Table 1) to protein samples produced new solution components that could be detected by sedimentation velocity analysis. Shown in Fig. 2 are c(M) plots for free 19-mer DNA, free proteins, and protein-DNA mixtures containing full-length WT-PARP1, or ∆CAT-PARP1, or ∆Zn1-Zn2-PARP1 proteins. At the concentrations tested, DNA mixtures with WT protein contained no material that co-sedimented with free DNA, and two c(M) peaks with apparent molecular weights of 117,560 ± 25,870 and 276,590 ± 40,350. The first peak overlaps substantially with the c(M) distribution of free protein, and the second is larger than that expected for a complex containing 2 protein monomers. The width of the c(M) distributions prevents us from determining whether the smaller peak corresponds to free protein (MW_predicted_ = 113,907) or a 1:1 protein-DNA complex (MW_predicted_ = 125,524) however, the simplest interpretation is that it represents free protein. Our current interpretation of the larger peak is that it corresponds to a mixture of complexes containing at least 2 protein molecules. The large width of these peaks, and the fact that c(M) does not reach the baseline between them, suggests that binding precursors and products are equilibrating on a time scale like that of sedimentation^48–50^. DNA mixtures with the ∆CAT protein contained only a trace of material co-sedimenting with free DNA, and two c(M) peaks with apparent molecular weights of 75,190 ± 8,495 and 174,760 ± 18,680. These are comparable to values for free protein (MW_predicted_ = 74,522) and complexes with ≥ 2 protein molecules (MW_predicted, 2:1_ = 160,661). Again, large peak-widths and the failure of the distribution to reach the baseline between them, suggest that components are equilibrating during sedimentation. DNA mixtures with the ∆Zn1∆Zn2 protein contained two c(M) peaks with apparent molecular weights of 27,050 ± 13,040 and 87,550 ± 15,230. The first value is larger than that of free DNA, although the c(M) envelopes overlap; the second is like that of free protein, but significantly less than that expected for a 1:1 complex (MW_predicted_ = 101,422). Our current interpretation of this pattern is that the first peak corresponds to DNA that migrates faster than unbound DNA, because it is equilibrating with protein, and the second peak depicts a sedimenting boundary with apparent molecular weight smaller than that of a 1:1 complex. Mobility like this would be observed if the DNA were equilibrating between complex and free states. A boundary that contains reaction components that equilibrate rapidly on the time scale of sedimentation does not correspond to a single species and is sometimes called a “reaction boundary”^50^. The formation of reaction boundaries during sedimentation complicates the estimation of binding stoichiometries and the estimation of affinities from the dependence of binding densities on protein concentration. We therefore turned to gel-electrophoretic mobility shift assays (EMSA), to take advantage of the kinetic stabilization of protein-nucleic acid complexes afforded by the gel environment^51–53^.

### Full length- and ∆CAT-PARP-1 proteins form multi-protein complexes with duplex 19-mer DNA

**Figure 3:**
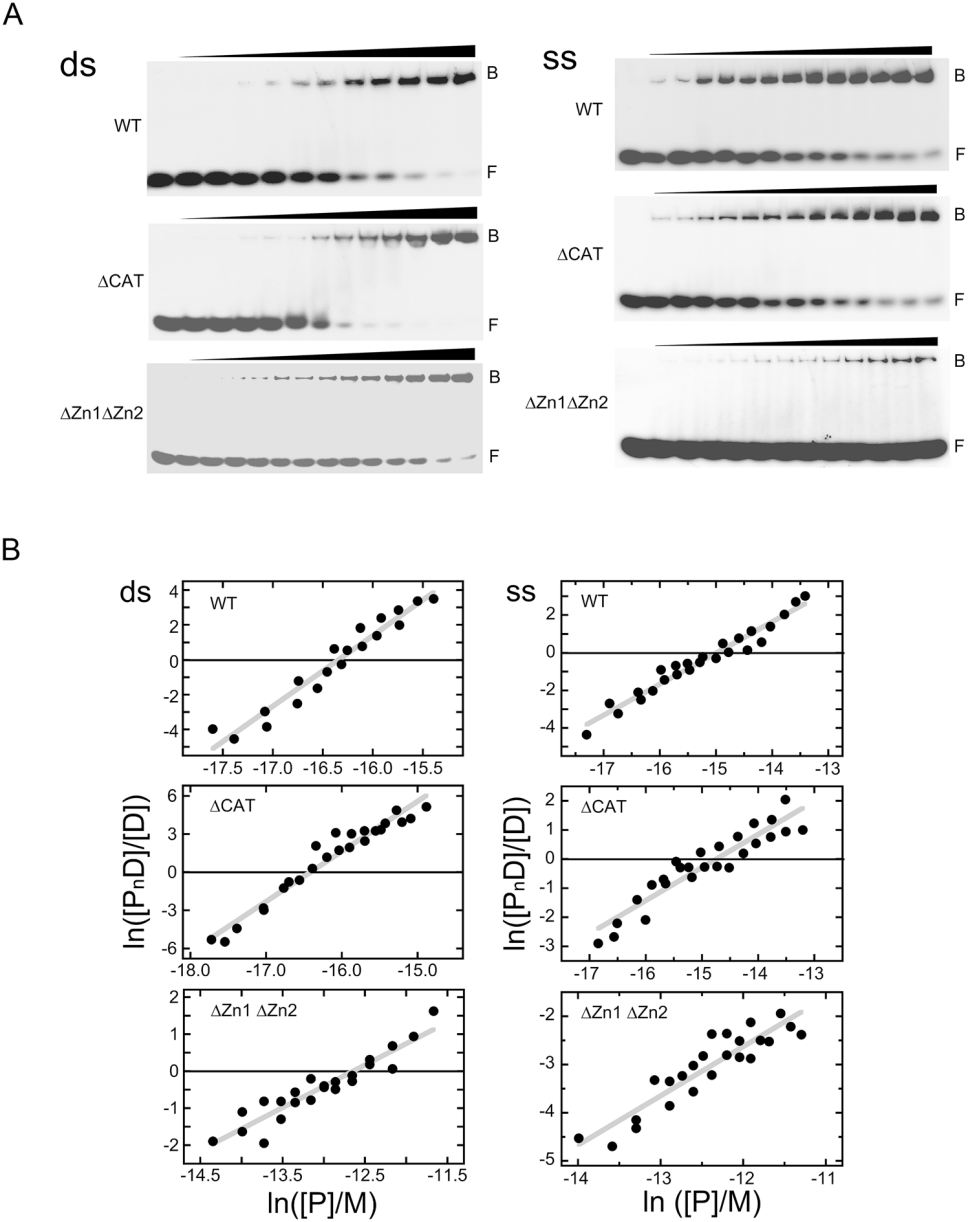
(**A)** Titration of double-stranded and single stranded 19-mer DNAs with WT-PARP1, ∆CAT-PARP1 and ∆Zn1∆Zn2-PARP1 proteins, detected by EMSA (Supplemental Figure S5 contains the full image and experimental replicates are in supplemental Figure S6). Samples formed with dsDNA contained 0.06 µM DNA and 0 – 0.25 µM WT-PARP1, or 0 – 0.3 µM ∆CAT-PARP1, or 0 – 10.9 µM ∆Zn1∆Zn2-PARP1. Samples formed with ssDNA contained 0.06 µM DNA and 0 – 1.5 µM WT-PARP1, or 0 – 1.63 µM ∆CAT-PARP1, or 0 – 12.4 µM ∆Zn1∆Zn2-PARP1. Buffer, incubation and electrophoresis conditions are described in Methods. (**B**). Analyses of interactions of WT-, ∆CAT- and ∆Zn1∆Zn2-PARP1 proteins with duplex 19-mer DNA (left) or single-stranded 19-mer DNA (right). Graphs show data from 2 independent titrations, plotted as described for Eq. (1). Note that scales for these graphs are not identical. Solid grey lines are least squares fits to each data set and the x-axis, at y = 0, is indicated by a solid black line. For dsDNA binding, these analyses returned n = 4.1 ± 0.2 for WT-PARP1, 3.9 ± 0.2 for ∆CAT-PARP1, and 1.2 ± 0.1 for the ∆Zn1∆Zn2 enzyme. For binding ssDNA, these analyses returned n = 1.8 ± 0.1 for WT-PARP1, 1.0 ± 0.1 for ∆CAT-PARP1, and 1.0 ± 0.1 for the ∆Zn1∆Zn2 enzyme. Monomer-equivalent association constants derived from these analyses are given in Table 3.

Titration of the ds19-mer DNA with full-length WT-PARP1 produces a species that migrates slowly during native electrophoresis in 8% polyacrylamide gels (Fig. 3A, top left). Complexes with similar electrophoretic mobilities were formed with the ∆CAT- and ∆Zn1∆Zn2-proteins (middle and lower panels, respectively). Sharp band boundaries, and the small mole fractions of dissociated DNA migrating between free and bound bands (mole fractions < 0.05, result not shown) indicate that these complexes are quite stable under the conditions of gel electrophoresis. This contrasts with their behaviors during sedimentation in the ultracentrifuge. Constant mobility shifts over the full ranges of fractional saturation are consistent with homogeneous binding mechanisms.

Graphs of ln [P_n_D]/[D] as functions of ln [P] for dsDNA binding by WT-PARP1, ∆CAT- and ∆Zn1∆Zn2- enzymes are shown in Fig. 3B (left). As described for Eq. (1), the slopes of these graphs give estimates of binding stoichiometries (n), while titration-midpoints give estimates of formation constants. Constant slopes, over the full ranges of each titration, are consistent with homogeneous binding mechanisms. These analyses returned n = 4.1 ± 0.2 for the binding of WT-PARP1, 3.9 ± 0.2 for the ∆CAT enzyme, and 1.2 ± 0.1 for the ∆Zn1∆Zn2 enzyme (Table 3). The stoichiometries found by EMSA for WT- and ∆CAT-enzymes are roughly twice those estimated by c(M) sedimentation analysis, and that for the ∆Zn1∆Zn2 enzyme is roughly 15% larger than that found by c(M). These results support our interpretation that the sedimentation velocity patterns that we observed were reaction boundaries and not true species. The formation of high-stoichiometry complexes from free DNA, in a single step and without accumulation of stoichiometric intermediates, is a hallmark of positively cooperative binding. The striking difference in stoichiometries for WT and ∆Zn1∆Zn2 enzymes suggest that deletion of Zn1 and Zn2 domains imposes a change of binding mechanism, either through modification of DNA-binding surfaces, or protein–protein interaction surfaces, or both.

**Table 3:** Stoichiometries and equilibrium constants for the binding of PARP proteins to single-stranded and duplex forms of the 19-mer DNA.

Enzyme and DNA tested	Stoichiometry^1^ (n)	(bp or nt) per protein for the complex^1^	Monomer equivalent association constant^1^ (K_mono_, M^−1^)	Cooperativity parameter^2^ (ω)
ds19-mer + WT-PARP1	4.1 ± 0.2	4.6 ± 0.2	1.2 ± 0.7 × 10^7^	82.6 ± 20.5
ss19-mer + WT-PARP1	1.8 ± 0.1	10.5 ± 0.5	3.3 ± 1.0 × 10^6^	24.7 ± 8.6
ds19-mer + ∆CAT-PARP1	3.9 ± 0.2	4.9 ± 0.2	1.3 ± 0.7 × 10^7^	139.9 ± 59.5
ss19-mer + ∆CAT-PARP1	1.0 ± 0.1	19 ± 1.7	1.14 ± 0.6 × 10^5^	–
ds19-mer + ∆Zn1∆Zn2-PARP1	1.2 ± 0.1	15.8 ± 1.2	3.1 ± 1.8 × 10^5^	–
ss19-mer + ∆Zn1∆Zn2-PARP1	1.0 ± 0.1	19 ± 1.7	2.9 ± 0.9 × 10^5^	–

^1^Values were obtained by evaluating the data shown in Figs. 3B, using Eq. (1). The monomer-equivalent association constants were obtained with the assumption of equipartition of binding free energies among all proteins, for which (K_mono_)^n^ = K’.

^2^Values were obtained by evaluating the data used in Fig. 3B, using Eq. (2). Analyses were carried out for systems in which stoichiometries were 2 or greater.

Equation 1 also provides a means of evaluating the formation constants (K’) of complexes (see above). Where n = 1, K’ is the association constant of the interaction. Were n > 1, K’ is the association constant for the overall assembly, taken as a single step. For complexes with n > 1, assumption of equipartition of binding free energies allows evaluation of monomer-equivalent association constants, K_mono_ = (K’)^1/n^. These are given in Table 3. Values from 3.1 × 10^5^ M^−1^ (∆Zn1∆Zn2) to 1.3 × 10^7^ M^−1^ (WT and ∆CAT enzymes), correspond to association free energies of -7.3 to -9.6 kcal/mol, in the mid-range of reported affinities for proteins binding to duplex DNAs^54^. The reduced affinity of the ∆Zn1∆Zn2 enzyme, compared to the WT and ∆CAT, reflects a role for zinc fingers 1 and 2 in DNA binding. This is in line with previous studies of Zn1 Zn2 binding to DNA^55^. However, the finding that the ∆Zn1∆Zn2 enzyme retains significant binding activity is consistent with other studies that other parts of the PARP1 structure also interact with DNA^4,5,11,14,16,56,57^.

### Stoichiometries and affinities differ for single stranded and duplex DNAs

PARP1 binds damaged and undamaged DNAs^58^ as well as RNA molecules^5,28,31,57–59^, so it was of interest to discover whether PARP1 interactions with single-stranded DNAs resembled those with duplex. Shown in Fig. 3A (right) and Supplemental Figures S5 and S6, are titrations of a single-stranded 19mer DNA with FL, ∆CAT and ∆Zn1∆Zn2 enzymes. All enzymes gave single mobility-shifted species with mobility decrements like those seen with duplex DNA. While the ∆Zn1∆Zn2 enzyme did not reach binding saturation in the concentration range tested, the data quality in all sets was good enough for analysis (Fig. 3B). Graphed as described for Eq. (1), these data returned n = 1.8 ± 0.1 for the binding of FL enzyme, n = 1.0 ± 0.1 for the ∆CAT enzyme, and n = 1.0 ± 0.1 for the ∆Zn1∆Zn2 enzyme. These values are significantly smaller than those found with duplex DNA of the same length (Table 3), suggesting a dramatic change in DNA binding mechanism with substitution of single-stranded substrate for duplex. In addition, both the ∆CAT and ∆Zn1∆Zn2 enzymes retained binding activity, while forming complexes with smaller stoichiometry than that formed by the full-length enzyme. Functional implications of these results will be discussed below. Because the binding stoichiometries of the proteins differ, the simplest comparison of affinities uses monomer-equivalent association constants (Table 3). On this basis, substitution of single-stranded DNA for duplex reduces the affinities of FL- and ∆CAT-enzymes, while that of ∆Zn1∆Zn2 is not significantly changed. These differences may suggest a role for the Zn1 and Zn2 domains in the partition of PARP1 between single-stranded and double-stranded DNA sites.

**Figure 4:**
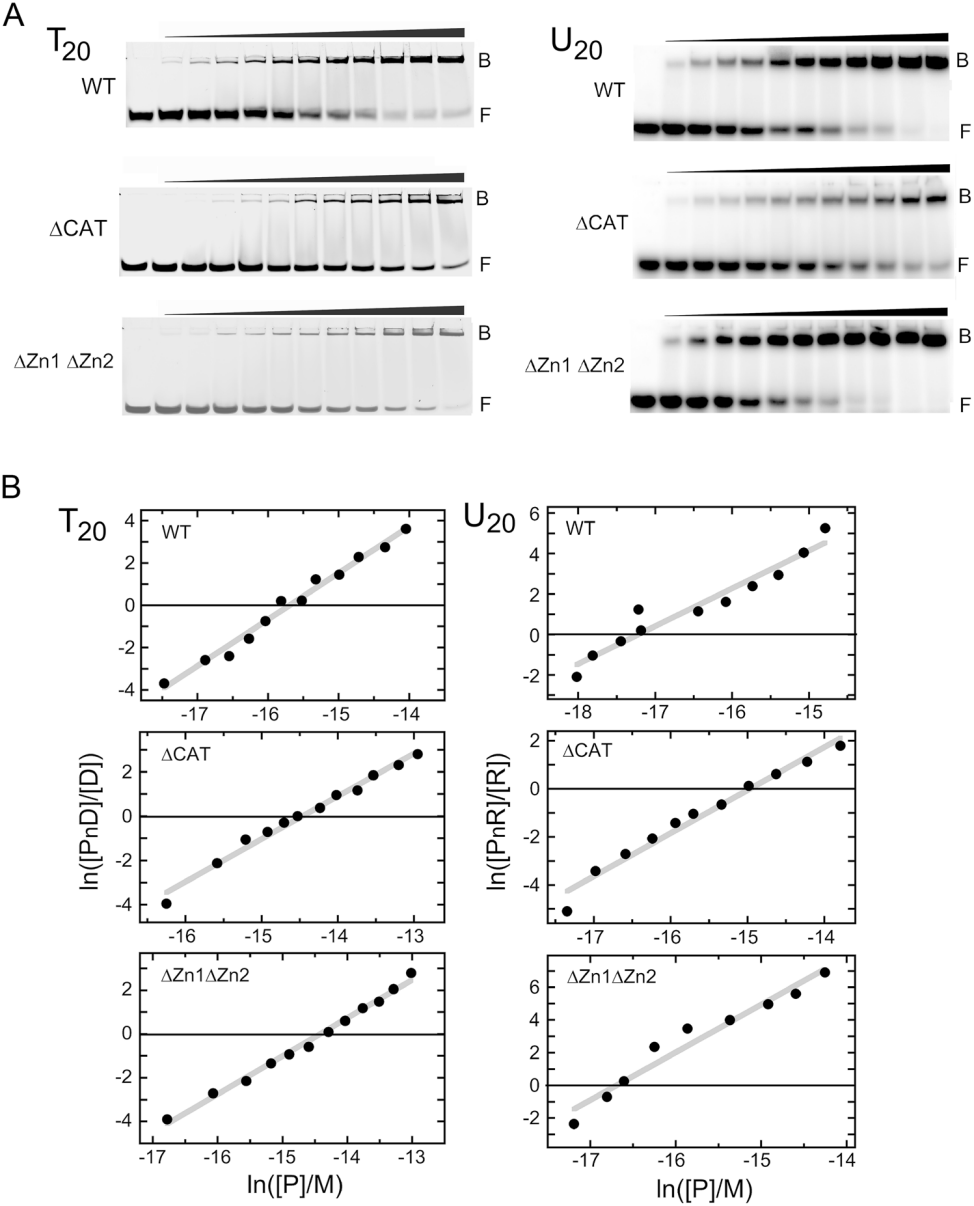
(**A)** Titration T_20_ and U_20_ with WT-PARP1, ∆CAT-PARP1 and ∆Zn1∆Zn2-PARP1 proteins, detected by EMSA (Supplemental Figure S7 contains the full image and experimental replicates are in supplemental Figure S8). T_20_ samples contained 0.06 µM T_20_ DNA and 0 – 2.34 µM WT-PARP1, or 0 – 7.74 µM ∆CAT-PARP1, or 0 – 7.09 µM ∆Zn1∆Zn2-PARP1. U_20_ samples contained 0.06 µM U_20_ RNA and 0 – 0.68 µM WT-PARP1, or 0 – 4.91 µM ∆CAT-PARP1, or 0 – 3.08 µM ∆Zn1∆Zn2-PARP1. Buffer, incubation and electrophoresis conditions are described in Methods. (**B**) Analyses of interactions of WT-, ∆CAT- and ∆Zn1∆Zn2-PARP1 proteins with single-stranded T_20_ DNA (left) or single-stranded U_20_ RNA (right). Graphs are plotted as described for Eq. (1). Note that scales for these graphs are not identical. Solid grey lines are least squares fits to each data set and the x-axis, at y = 0, is indicated by a solid black line. For T_20_ DNA binding, these analyses returned n = 2.2 ± 0.1 for WT-PARP1, 1.9 ± 0.1 for ∆CAT-PARP1, and 1.7 ± 0.1 for the ∆Zn1∆Zn2 enzyme. For U_20_ RNA binding, analyses returned n = 1.9 ± 0.2 for WT-PARP1, 1.8 ± 0.1 for ∆CAT-PARP1, and 2.9 ± 0.2 for the ∆Zn1∆Zn2 enzyme. Monomer-equivalent association constants derived from these analyses are given in Table 4.

PARP1 binds preferentially to sites of DNA damage^60^, sites of chromatin remodeling^59,61^, and some promoter sequences^22,62,63^. However, the roles of DNA sequence-specific and/or structure-specific interactions in its binding preferences remain to be determined. As a step in this process, we examined PARP1 binding to T_20_ (5’ labeled with cy3), a DNA of uniform base composition with a low propensity to form base-paired secondary structures. Shown in Fig. 4A (left), and Supplemental Figures S7-S8, all proteins formed strongly-mobility-shifted species with this DNA, with some material barely penetrating the gel. This is sometimes seen when complexes tend to aggregate, and here those species were quantitated as part of the bound fraction. Binding analysis carried out as described for Eq. (1) returned stoichiometries of 2.2 ± 0.1 for WT-PARP1, 1.9 ± 0.1 for ∆CAP-PARP1, and 1.7 ± 0.1 for ∆Zn1∆Zn2-PARP1. As before, the formation of these complexes without accumulation of 1:1 intermediates is evidence of positively-cooperative binding. The WT-PARP1 protein forms 2:1 complexes with ss19mer and T_20_ DNAs. However, the stoichiometries of complexes formed by ∆CAT-PARP1 are different (n = 1.0 ± 0.1 for the ss19mer but n = 1.9 ± 0.1 for T_20_). The ∆Zn1∆Zn2-PARP1 also gives different stoichiometries with ss19mer and T_20_ (n = 1.0 ± 0.1 for the ss19mer but n = 1.7 ± 0.1 for T_20_). While the small increase in DNA contour length (20nt as opposed to 19nt) might allow the binding of an additional protein monomer, another potential source of difference is sequence-specific interaction, since each ssDNA offers base-contacts that are not available in the other. However, we favor the first mechanism, because binding affinities on T_20_ DNA are, in fact, greater than those for the ss19mer. This suggests that feature(s) relevant to binding may be augmented in the longer polymer. Possibilities include better accommodation of proteins due to the increased length of T_20_, stronger interactions with oligo dT than with ss19mer sequences, and for the ∆CAT and ∆Zn1∆Zn2 enzymes (which form 2:1 complexes on T_20_), cooperative interactions that are not possible in the 1:1 complexes formed with the ss19mer.

### RNA binding

PARP1 binds RNA and important cellular functions have been attributed to its interactions with that polymer^5,29,64^. Since many secondary structures are available in natural RNAs, it is likely that PARP1-RNA complexes have a parallel variety in structure, stoichiometry, and stability. Here we examined binding to U_20_, which provides binding sites of uniform base composition and has a low propensity to form base-paired secondary structures. Shown in Fig. 4A (right) and Supplemental Figures S7-S8, all proteins formed single mobility-shifted species with this RNA. Binding quantitation and analysis as described for Eq. (1) returned stoichiometries of 1.9 ± 0.2 for WT-PARP1, 1.8 ± 0.1 for ∆CAT-PARP1, and 2.9 ± 0.2 for ∆Zn1∆Zn2-PARP1 (Fig. 4B). The first two values are like those found with T_20_, raising the possibility that WT- and ∆CAT-proteins bind similarly with U_20_ and its DNA analogue. The U_20_-binding of the ∆Zn1∆Zn2 protein contrasts with those of WT- and ∆CAT-molecules. The larger stoichiometry (2.9 ± 0.2) implies a smaller contour length per protein (~ 6.9nt/protein as opposed to ~ 10; Table 4) and suggests a different packing mechanism for this protein when it binds RNA. In this context, it is especially interesting that cooperative binding is preserved.

**Table 4:** Stoichiometries and equilibrium constants for the binding of PARP proteins to single-stranded T(20) and U(20) nucleic acids.

Enzyme and DNA tested	Stoichiometry^1^ (n)	nt/protein^1^	Monomer equivalent association constant^1^ (K_mono_, M^−1^)	Cooperativity parameter^2^ (ω)
T(20) + WT-PARP1	2.2 ± 0.1	9.1 ± 0.4	1.3 ± 1.0 × 10^7^	51.4 ± 10.1
U(20) + WT-PARP1	1.9 ± 0.2	10.5 ± 1.0	2.1 ± 1.4 × 10^8^	67.3 ± 10.8
T(20) + ∆CAT-PARP1	1.9 ± 0.1	10.5 ± 0.5	3.3 ± 2.6 × 10^6^	49.0 ± 12.9
U(20) + ∆CAT-PARP1	1.8 ± 0.1	11.1 ± 0.6	6.6 ± 3.4 × 10^6^	86.1 ± 20.3
T(20) + ∆Zn1∆Zn2-PARP1	1.7 ± 0.1	11.7 ± 0.6	2.5 ± 1.6 × 10^6^	56.7 ± 14.4
U(20) + ∆Zn1∆Zn2-PARP1	2.9 ± 0.2	6.9 ± 0.4	9.1 ± 7.4 × 10^7^	104.3 ± 31.1

^1^Values were obtained by evaluating the data shown in Figs. 5 and 6, using Eq. (1). The monomer-equivalent association constants were obtained with the assumption of equipartition of binding free energies among all proteins, for which (K_mono_)^n^ = K’.

^2^Values were obtained by evaluating the data used in Fig. 4B, using Eq. (2).

### Evaluation of cooperativity

**Figure 5:**
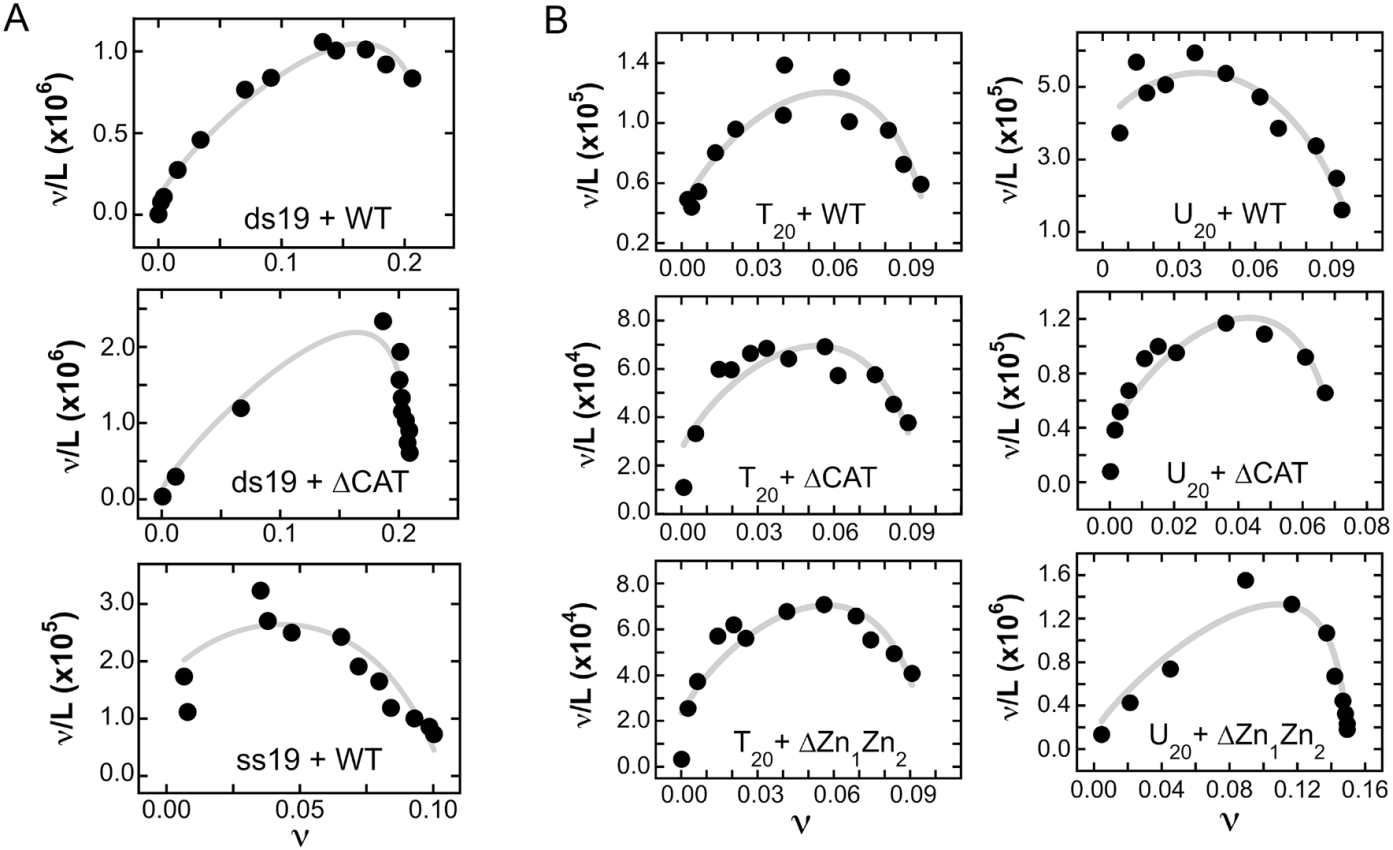
(**A**) Scatchard plots for wild type PARP1 and ∆CAT PARP1 with single-stranded and duplex 19mer DNAs. The binding data is from the EMSA experiments shown in Fig. 4A. The smooth curves are fits of Eq. (2) to the data. The cooperativity parameters returned by these fits are shown in Table 3. Note that the scales for each graph differ from those of other graphs. (**B**) Scatchard plots for wild type, ∆CAT and ∆Zn1∆Zn2 PARP1 proteins with single-stranded T_20_ DNA and U_20_ RNA. The binding data is from the EMSA experiments shown in Fig. 5A. The smooth curves are fits of Eq. (2) to the data. The cooperativity parameters returned by these fits are shown in Table 4. Note that the scales for each graph differ from those of other graphs.

Shown in Fig. 5 are Scatchard plots of data for binding duplex and single-stranded 19mer DNAs (panel A) and for binding T_20_ DNA and U_20_ RNA molecules (panel B). The concave downward trends of these plots indicate positively cooperative binding^45^. Accordingly, we used the short-lattice version of the McGhee-von Hippel relation (Eq. 2) to estimate cooperativity values for interactions in complexes containing two or more proteins. Values returned by these analyses are given in Tables 3 and 4. The range of values over all complexes with n ≥ 2 is approximately 24 ≤ $\omega$  ≤ 140. These are modest compared to some values reported for single-stranded binding proteins ($\omega$ ≥ 10^3^)^65,66^ but other molecular systems give values in the range that we report here (c.f.,^67,68^). Even these modest values are sufficient to give single-step binding transitions in which free nucleic acid substrates are converted into multi-protein complexes without significant accumulation of stoichiometric intermediates. This characteristic will be discussed more below.

## Discussion

PARP1 is a multi-functional enzyme that binds DNA^57,58,69,70^, RNA^5,29,31,64^ and many proteins^20,21,71^. PARP1 autoPARylates itself^72,73^ as well as modifying several substrates including DNA^14,74–76^, histones^3,26,69,77^, DNA repair proteins, and RNA splicing factors^20,21,71^. As a result, PARP1 is positioned at the intersection of important cellular pathways, including transcription, DNA repair and RNA maturation, where it has the potential to contribute to the coordination of these processes. This multiplicity of important functions justifies thorough characterization of its interactions with binding partners, substrates and products, and this effort is well underway^5,31,57–59^.

PARP1 is a modular enzyme, in which individual domains retain some of their functions in isolation or in the context of a subset of other PARP1 domains. This feature has been valuable for the assignment of functions to individual domains and an aid to crystallographic studies where flexible coupling between domains is sometimes problematic^78,79^. However, it can lead to an incomplete understanding of a function to which several domains contribute^80^. One such function is nucleic acid binding, where several PARP1 domains^10,14,16^ such as the Zn1 and Zn2 zinc-finger domains^55^*,* Zn3^11,14,16,56^, WGR^4^ and the BRCT domain contribute to PARP1-DNA binding^57^. Another is protein multimerization, where two different domains may form the interaction interface. This idea is supported by recent studies of PARP1 multimerization with longer dsDNA to drive condensation in vivo^81^. Effects of changing the structural context of domains are evident in our comparison of the DNA binding stoichiometries of WT-PARP1 and the ∆Zn1∆Zn2 mutant with, for example, the double-stranded 19mer (Table 3). We believe that the functional effects of changing the structural context of domains account for most, if not all, of the binding differences that we have observed between WT, ∆CAT and ∆Zn1∆Zn2 proteins.

Our results show that WT, ∆CAT and ∆Zn1∆Zn2 proteins are monomers under our solution conditions, in the absence of nucleic acids. This is consistent with results of some previous studies^13,14,81^ although others report that free PARP is dimeric^17,32,55,82^. This difference in association state is probably due to differences in enzyme preparation or in the solution conditions under which measurements were made. Whatever the source, it emphasizes the importance of establishing the quaternary state of PARP1 as a part of studies of binding or enzymatic activity. Evidence that PARP1 is monomeric under our conditions argues against mechanisms in which it binds nucleic acids as a pre-formed dimer (or higher multimers in the reactions that form 3:1 and 4:1 complexes described in results). The model that we currently favor is one in which PARP1 binds first as a monomer, then cooperative interactions favor the formation of higher-stoichiometry complexes, if permitted by the structure of the nucleic acid substrate. At the start of a titration, all proteins bind isolated sites. After this initial phase, incoming proteins distribute between isolated sites and those adjacent to bound proteins. Positive cooperativity enhances the affinity of incoming proteins for sites adjacent to bound proteins. This difference in affinity ensures that adjacent sites fill up before isolated ones, saturating the first nucleic acids bound before significant binding to isolated sites occurs. Such a mechanism might lead to the formation of cooperative assemblies of PARP1 in vivo.

Here we have examined the binding of wild-type PARP1 and two truncation mutants to short nucleic acids (blunt ended with mixed nucleotides except for T_20_ and U_20_ – see Table 1), with results that shed light on their binding selectivities and protein–protein interactions. Cooperative binding is the most striking feature of these interactions. This is evidenced by the formation of complexes with stoichiometry ≥ 2 without prior accumulation of 1:1 complexes. All proteins tested were capable of cooperative binding. This was surprising, as the Zn-finger domains missing in the ∆Zn1∆Zn2 mutant are known DNA binding sites^55^, while the catalytic domain that is missing in ∆CAT is implicated in protein–protein interactions necessary for ADP-ribosylation of substrate proteins, including other molecules of PARP1^19–21^. These results show that neither the Zn1Zn2 region nor the CAT domain alone are essential for nucleic acid binding or cooperativity. Since the effects of removing the Zn1Zn2 and CAT domains were tested separately, it remains possible that the presence of the CAT domain complements the loss of Zn1Zn2 functions, and vice-versa. However, these results are consistent with results suggesting that regions other than Zn1Zn2 contribute to PARP1-DNA binding^14,57,83^ and regions other than the CAT domain contribute to binding cooperativity. It has been shown that PARP1 adopts a collapsed conformation for substrate recognition^9,84^, potentially using various domains or combinations for multifunctionality. In DNA break recognition, domains such as Zn1, Zn2, Zn3, WGR, and catalytic domains converge at the damaged site as reflected in partial structures obtained with PARP1 fragments bound to a double-stranded DNA break^14^, and to nicked DNA^6^. This mechanism may occur in the recognition of dsDNA ends, recapitulating ds breaks and/or structure of the DNA, however further studies are needed to explore this idea. In addition, it has been argued that depending on the context, PARP1 may bind as a monomer or a dimer. Activating partners for PARP1 range from damaged/structured DNA^85,86^, nuclear proteins^62,85–87^ to post-translational modifications^88^, thus influencing its binding mode in non-active and activated states. In the absence of DNA damage, cooperativity with DNA, RNA, itself, and other proteins might help PARP1 in recognizing non-damaged DNAs serving as a hub in recruiting proteins, different nucleic acids for gene expression. A recent study showed PARP1 multimerization binding to dsDNA, highlighting the critical role of protein–protein interactions between two PARP1 molecules in bridging DNA molecules^81^.

The monomer-equivalent association constants that we have found lie in a range (10^5^–10^8^ M^−1^) that is typical of many protein-nucleic acid interactions. However, the differences in affinity that accompany substitution of one nucleic acid substrate for another are revealing. Thus, for the WT protein, substituting ss19mer for the duplex reduces affinity by ~ fourfold, but the same substitution for the ∆CAT protein reduces affinity by ~ 110-fold. Intriguingly, ∆CAT forms a 3.9:1 complex with ds19mer and only a 1:1 complex with ss19mer. This suggests that this double stranded DNA with mixed nucleotides might provide the surface needed for cooperativity by this mutant. In a similar vein, we found that the WT enzyme bound U_20_ RNA about 15-times more tightly than T_20_ DNA, while the ∆Zn1∆Zn2 mutant enzyme bound U_20_ RNA at most 40-times more tightly than T_20_ DNA, suggesting that domains other than Zn1Zn2 participate in RNA binding. A similar conclusion, based on different observations has been published^5^. The ∆CAT mutant protein showed a reduced difference in affinity for U_20_ and T_20_, with only two-fold greater affinity for U_20_ compared to T_20_ (Table 4). These results suggest that protein structures that play a role in distinguishing RNA from DNA, or possibly uridine from thymidine, have been modified by the mutations that excised the CAT domains.

All the proteins tested here were capable of binding cooperativity when the substrate was single-stranded T_20_ or U_20_. For the ∆CAT and ∆Zn1∆Zn2 enzymes, these results indicate that neither of these domains is a unique functional determinant of cooperative binding. Thus, it is striking that both ∆CAT and ∆Zn1∆Zn2 enzymes are limited to 1:1 complexes when the binding substrate is the slightly shorter ss19mer DNA. This might reflect sequence-specific differences in binding mechanism, or it might reflect a difference in the ability of these enzymes to bind 19 nt and 20 nt nucleic acids. We note that both WT and ∆CAT enzymes are capable of forming 4:1 complexes on ds19mer templates (corresponding to ~ 5 bp/protein), and ∆Zn1∆Zn2 forms a 3:1 complex on U_20_ (corresponding to ~ 6.6 nt/protein). Thus, all proteins are capable of tighter packing than that occurring in the 1:1 complexes. The roles of sequence and nucleic acid length in regulating the packing interactions of PARP1 are important questions for the future.

Cooperativity parameters, along with their standard deviations, were derived by fitting Eq. (2) to binding data. Experiment-specific values are detailed in Tables 3 and 4. Our cooperativity measurements exhibit overlapping error ranges, typically around ~ 30% of the central values. This prevents us from detecting small differences that may exist between different proteins or between different binding substrates. With that caveat, it is striking that similar cooperativity values are found in dense complexes (e.g., WT and ∆CAT proteins with double stranded 19mer; stoichiometry 4:1, average binding site sizes ~ 4.7 bp/protein) and more diffuse ones (e.g., proteins binding T_20_, stoichiometry ~ 2:1, average site sizes of ~ 10nt/protein). We do not know the geometry of these complexes, and so cannot account for this similarity. There are two non-mutually exclusive possibilities: 1. It is possible that PARP1 binds to the ends of the blunt-ended dsDNA reflecting dsDNA break recognition. Such a possibility was recently supported by Chappidi and colleagues^81^, showing that PARP1 binds to blunt-ended dsDNA and facilitates bridging between DNA fragments via PARP1-PARP1 interactions (positive cooperativity), crucial for condensing both PARP1 and DNA. 2. Another possibility is that cooperativity allows the sequential binding of PARP1 molecules along the DNA (binding site size as calculated in Tables 3 and 4). A better accounting for the contributions of cooperative binding to the stability of PARP1 complexes will have to wait for more detailed information about the distribution of these protein molecules on these DNA and RNA substrates.

Several studies have shown that PARP1 binds nicks, abasic sites and ends as a monomer^6,35,89^. While other studies using a Zn1-Zn2 domain fragment indicate that PARP1 binds to 3′ overhang DNA as a dimer^12^. These apparently conflicting results could reflect the use of different mutant proteins, or solution conditions or nucleic acid substrates. On the other hand, PARP1 may still bind as a monomer with subsequent cooperative binding between PARP1 molecules to ensure stable DNA binding. PARP1 was shown to bridge undamaged DNAs in loop stabilization^58^ and PARP1 can also move along undamaged DNA via the diffusion-limited ‘monkey-bar’ mechanism^9^. While these studies do not directly show that PARP1 binds as a monomer, they suggest cooperativity in PARP1 mode of binding. Additionally, PARP1's activity as a monomer^14,90^ or dimer^11,91,92^ can lead to PARylation in cis or trans. However, our studies do not assess PARP1's activity.

The ability to bind cooperatively in vitro may reflect an activity of the enzyme in vivo. The data presented here leads us to speculate that functional PARP1 complexes may contain ≥ 1 molecule of the enzyme. This idea is supported by the recent paper of Chappidi showing condensation of PARP1 molecules with dsDNA in the formation of condensates^81^. In addition, multimerization of PARP1 could enhance the occupancy of available DNA and RNA sites and contribute to self- and hetero-PARylation. Thus, the binding cooperativities of PARP1 may play roles in the delivery of poly ADP-ribosylation activity to genomic sites where this post-translational modification can play a regulatory role in gene expression.

### Supplementary Information

Supplementary Information.

## Data Availability

All data generated or analyzed during this study are included in this manuscript or supplementary information files.
