# Supplementary material for: Cooperative nucleic acid binding by Poly ADP-ribose polymerase 1
Source: Sci Rep. 2024 Mar 29;14:7530. doi: 10.1038/s41598-024-58076-w (PMC10980755; doi:10.1038/s41598-024-58076-w)
Supplement: Supplementary file 1 — Supplementary Information. [file 41598_2024_58076_MOESM1_ESM.docx]

Cooperative Nucleic acid Binding by Poly ADP-Ribose Polymerase

Running Title: PARP Binding Cooperativity

Manana Melikishvili^1^, Michael G. Fried^2^*, and Yvonne Fondufe-Mittendorf^1^*

1. Department of Epigenetics, Van Andel Institute, Grand Rapids MI 49503
2. Center for Structural Biology, Department of Molecular and Cellular Biochemistry, University of Kentucky, Lexington, KY 40536

*Address correspondence to: Michael G. Fried or Yvonne Fondufe-Mittendorf,

E-mail: michael.fried@uky.edu or y.fondufemittendorf@vai.org

**Supplemental Figure S2: Analysis of the PARP1-FL-WT and mutant through the purification steps. Supplemental Figure 1A, D, G: Protein** analysis from the nickel column purification step. Lane 1: Supernatant from cell lysate; Lane 2: Flow through from the nickel column; Lane 3: Pre-stained protein markers; Lane 4: Nickel column washing with low-salt wash buffer; Lane 5: Nickel column washing with high-salt wash buffer; Lane 6: Elution from nickel column; Lanes 7-9: Protein elution from HiTrap Heparin HP column by stepwise gradient of salt concentration.

**Supplemental Figure 1B, E, H:** Western blot analysis of the samples in A of FL-WT-PARP1 (B); samples in D of ΔZn1ΔZn2-PARP1 (E) and samples in G of ΔCAT-PARP1 (H).

**Supplemental Figure 1C, F, I**: Analysis of fractions after gel filtration chromatography from WT-FL-PARP1 (G); ΔZn1ΔZn2-PARP1 (H) and ΔCAT-PARP1 (I). Lane 1- Pre-stained protein marker; Lane 2: concentrated protein pooled after Heparin column; Lanes 3 -10: Peak-containing fractions after Sephacryl S200 column - fractions from C (lanes 6-7), F (Lanes 8-9) and I (lanes 6-7) were pooled for experiments and shown in Figure 1. *N. B: Protein samples were run on 4-12% gradient SDS-PAGE gels and the top 4% staking gel was cut off before Coomassie staining and/or western blot analysis. Protein samples were run on two gels, one for Coomassie staining and the other for Western blot analysis. The far-left panel of gels were stained with Coomassie, and the middle panel with the same samples was western blotted with PARP1-N antibody. Shown are representative images of full-length blots, revealing the gel edges and as well as all potential molecular weight markers of the pre-stained multicolor broad range protein ladder (ThermoFisher Scientific - cat #26634), visible on both Coomassie and Western blot images, including the gel edges.

Supplemental Figure S2: Full image of Figure 1C both Coomassie and Western blot analysis of purified proteins. The regions cut are boxed and showed as Figure 1C.

**Supplemental Figure 3: Circular dichroism spectra of WT-FL-PARP1 and mutant PARPs used in this study**. The spectra were obtained at 4°C using a Jasco J-815 spectropolarimeter (JASCO analytical instruments) and a 1 mm path length CD cell (Starna Scientific). The protein concentrations were 2.5 µM in the buffer: 10 mM Tris-HCl, pH 8.0, 75 mM NaCl, 0.1 mM TCEP. The final spectra for proteins represent the averages of three scans. All data were background corrected and have been converted from raw ellipticity to molar ellipticity.

**Supplemental Figure S4: Analysis of the ^32^P-labelled single stranded DNA and double stranded DNAs used in experiments.** DNAs were run on an 11% Native PAGE gel (bis-acrylamide to acrylamide ratio of 1/75 in 1XTBE). Lane 7: ss-19mer-DNA (0.1 µM); Lane 8: ss-19mer-DNA (0.06 µM); Lane 9: ds-19mer-DNA (0.11 µM). Since we show the full gel, we have other DNAs not used in study as indicated (Lanes 1 - 6 & 10 - 13). Top gel is from the boxed region of the lower full gel, showing the DNAs used in the study.

Supplemental Figure S5: Full images showing the top of the gel and lanes from which Figure 3A was cut. Images were detected via scanning of storage phosphor screens, enabled by the detection of ^32^P-labeled DNAs. First Lane is DNA alone without proteins. The top of the gels (or wells), xylene cyanol dye and bromophenol blue dye locations are marked by red, green, and blue arrows respectively. *****Positions of the dyes were marked with radioactive sharpie before exposing to the screen.

Supplemental Figure S6: Replicates of experiments depicted in Figure 3A, showing the top of the gels and lanes. Images were detected via scanning of storage phosphor screens, enabled by the detection of ^32^P-labeled DNAs. First Lane is DNA alone without proteins. The top of the gels (wells), xylene cyanol dye and bromophenol blue dye locations are marked by red, green, and blue arrows respectively. *****Positions of dyes were marked with radioactive sharpie before exposure to the screen.

Supplemental Figure S7: Full images showing the top of the gel and lanes from which Figure 4A was cut. Images on the right panel were detected via scanning of storage phosphor screens, facilitated by ^32^P-labeled DNAs. Meanwhile, images on the left panel were scanned on Typhoon using a laser line suitable for Cy3 detection. The top of the gels (wells), xylene cyanol dye, bromophenol blue dye and orange G dye locations are marked by red, green, blue, and orange arrows respectively. To ensure visibility, only Orange G dye, which migrates faster than Cy3-dT_20_, was added to these DNA samples. To mark the gel run, a mixture of all three dyes (bromophenol blue, xylene cyanol and orange G, was added to the first well which is sample free and used as a marker of gel run (gels on the left). *****Positions of dyes were marked with radioactive sharpie before exposure to screen.

Supplemental Figure S8: Additional replicates of experiments from Figure 4A displaying full gel images with lanes. Images in the right panel were detected via scanning of storage phosphor screens, enabled by ^32^P-labeled DNAs. Conversely, images in the left panel were scanned on Typhoon using a Cy3 detection-compatible laser line. Gel tops (wells), xylene cyanol dye, bromophenol blue dye, and orange G dye locations are denoted by red, green, blue, and orange arrows, respectively. To enhance visibility, only Orange G dye, which migrates faster than Cy3-dT_20_, was included in these DNA samples. A mixture of all three dyes (bromophenol blue, xylene cyanol, and orange G) was added to the first well, devoid of samples, to mark the gel run (visible in the left gels). *Positions of dyes were marked with radioactive sharpie before exposing to screen.
